# Hydrodynamic and heat transfer analysis of dissimilar shaped nanoparticles-based hybrid nanofluids in a rotating frame with convective boundary condition

**DOI:** 10.1038/s41598-021-04173-z

**Published:** 2022-01-10

**Authors:** Muhammad Ramzan, Nazia Shahmir, Hassan Ali S. Ghazwani, Kottakkaran Sooppy Nisar, Faizah M. Alharbi, I. S. Yahia

**Affiliations:** 1 Department of Computer Science, Bahria University, Islamabad, 44000 Pakistan; 2 Department of Mechanical Engineering, Faculty of Engineering, Jazan University, Jazan, 45124 Kingdom of Saudi Arabia; 3 Department of Mathematics, College of Arts and Sciences, Prince Sattam Bin Abdulaziz University, Wadi Aldawaser, 11991 Saudi Arabia; 4 Common First Year Deanship, Umm Al-Qura University, Makkah, Saudi Arabia; 5 Laboratory of Nano-Smart Materials for Science and Technology (LNSMST), Department of Physics, Faculty of Science, King Khalid University, P.O. Box 9004, Abha, Saudi Arabia; 6 Research Center for Advanced Materials Science (RCAMS), King Khalid University, P.O. Box 9004, Abha, 61413 Saudi Arabia; 7 Nanoscience Laboratory for Environmental and Biomedical Applications (NLEBA), Semiconductor Laboratory, Department of Physics, Faculty of Education, Ain Shams University, Roxy, Cairo, 11757 Egypt

**Keywords:** Software, Mechanical engineering

## Abstract

Solar thermal systems have low efficiency due to the working fluid's weak thermophysical characteristics. Thermo-physical characteristics of base fluid depend on particle concentration, diameter, and shapes. To assess a nanofluid's thermal performance in a solar collector, it is important to first understand the thermophysical changes that occur when nanoparticles are introduced to the base fluid. The aim of this study is, therefore, to analyze the hydrodynamic and heat characteristics of two different water-based hybrid nanofluids (used as a solar energy absorber) with varied particle shapes in a porous medium. As the heat transfer surface is exposed to the surrounding environment, the convective boundary condition is employed. Additionally, the flow of nanoliquid between two plates (in parallel) is observed influenced by velocity slip, non-uniform heat source-sink, linear thermal radiation. To make two targeted hybrid nanofluids, graphene is added as a cylindrical particle to water to make a nanofluid, and then silver is added as a platelet particle to the graphene/water nanofluid. For the second hybrid nanofluid, CuO spherical shape particles are introduced to the graphene/water nanofluid. The entropy of the system is also assessed. The Tiwari-Das nanofluid model is used. The translated mathematical formulations are then solved numerically. The physical and graphical behavior of significant parameters is studied.

## Introduction

Solar energy has been considered a significant source of energy for many years due to the huge amounts of energy that are made freely available when modern technology is used to collect it. The planet receives a total of $4 \times 10^{15} {\text{m\,W}}$, which is almost 200 times more than what is normally used. Solar thermal energy is a technique of absorbing the sun's energy and converting it into thermal energy^1^. There are plates where the collectors are immersed in a solution of water (H_2_O) and Ethylene Glycol, which transmits heat to the solution. On the other hand, their main drawback is that these traditional liquids have poor thermal efficiency when moving due to their lack of good thermal transfer characteristics. The addition of nanometer-sized particles in fluid changed the thermal characteristics of the base fluid. The main significant thermophysical properties impacting nanofluids' convective heat transfer performance are their thermal conductivity and dynamic viscosity^2^. Results of the experiments demonstrate that the thermal conductivity and dynamic viscosity of nanofluids are dependent on the size, particle shape, and the kind of base liquid and operating temperature of the nanofluid^3–6^. An increase in nanoparticle concentration can result in an enhancement in thermal conductivity and viscosity, whereas an increase in nanoparticle size can result in either an increment in thermal conductivity while lowering nanofluid viscosity^7^. Natarajan and Sathish^8^ investigated the use of carbon nanotubes to enhance working liquid thermal conductivity and hypothesized that using CNT-based nanofluids as a heat transfer medium might improve the performance of standard solar water heaters. In the collector's solar system, Stalin et al.^9^ employed CeO_2_/H_2_O nanofluid to assess the impact on the efficiency of the systems of adding nanostructures. The observations show that in the event of the use of the nanofluid, the collector efficiency may be enhanced by 21.5%.

Even greater improvement in the thermophysical characteristics of nanofluids may be achieved by the use of hybrid nanomaterials, which are composed of several materials with nanoscale dimensions^10^. The usage of hybrid nanofluids in the solar collector and the improvement of performance have increased in literature. Hybrid nanofluids can therefore improve thermal conductance throughout a range of temperatures at lower concentrations. The hybrid nanofluids CuO-MWCNTs were experimentally produced by Qu et al.^11^ to collect direct solar thermal energy. Akilu et al.^12^ examined the thermophysical characteristics of SiO_2_–CuO/C hybrid nanofluid-based glycerol and EG combination, and observed a 26.8 percent increased thermal conductivity. The tests showed the hybrid an appealing HTF for transporting solar energy. In the solar vapor production system, Ghafurian et al.^13^ used graphene oxide and water nanofluid and compared them with water as a working liquid. The overall efficiencies of systems in the same conditions were found to be 54% and 78.9% for pure water and nanofluid. Using a 3-D homogeneous mixture model, Alazwari and Safaei^14^ scrutinized the influence of a Baffle layout and a hybrid nanofluid on the thermal performance of a shell and tube heat exchanger. The hybrid nanofluid was discovered to have the potential to be used in a shell and tube heat exchanger. However, pumping power is increased, which may be adjusted by rearranging the heat exchanger arrangement, nanoparticle size, and base fluid type. Anitha et al.^15^ studied the performance of thermal and energy management potentials of γ-AlOOH hybrid nanofluids for employment inefficient heat exchanger systems. The pumping power of TiO_2_-γ-AlOOH/EG Hybrid nanofluid is found to be more than that of TiO_2_-γ-AlOOH/H_2_O hybrid nanofluid. These sources^16–24^ contain additional research and experimental work on mono/hybrid nanofluid flow with practical applications.

Convective heat transfer is extremely essential in procedures involving high temperatures. For example, thermal collectors, nuclear power plants, thermal energy storage, and so on. Convective heat transfer is extremely important in procedures involving high temperatures. For example, gas turbines, nuclear power plants, thermal energy storage, and so on. Aziz^25^ pioneered the use of convective surface boundary conditions to study boundary layer flow in the classic Blasius issue on a flat surface. He investigated the existence of a similarity solution for laminar thermal boundary layer flow on a flat plate under convective boundary conditions. Nasrin et al.^26^ scrutinize the heat transport processes of a flat plate solar collector equipped with various nanofluids using numerical simulations, and the results were published. Collector efficiency was higher for Ag/water than for Cu/water, with an increase of around 13 percent for Ag/water and an increase of 8 percent for Cu/water. Shehzad et al.^27^ used Brownian and thermophoresis diffusion effects to examine the convective heat transfer characteristics of a nanoliquid flow in a wavy channel. Considerable work has been expended in studying the effects of convective boundary conditions in solar application in various directions^28–33^.

Numerous applications in industry and technology are made possible by the flow that occurs in a spinning system. The flow of liquid in a spinning system is a completely natural phenomenon. During the rotation of the fluid, the molecules of the liquid clash with one another, resulting in changes in velocity, volume, density, and other properties. In reality, as soon as the fluid begins to flow, the internal rotation of the fluid increases. This rotation can be minimized, but it cannot be eliminated. Attia et al.^34^ investigated the flow of an electrically conducting viscous liquid between two (horizontal) parallel plates with changing viscosity in the presence of a variable voltage. Greenspan^35^ has also conducted detailed research on the flow of liquid in a rotating system, which may be found here. An additional study was carried out by Vajravelua and Kumar^36^, who examined magneto hydrodynamically (viscous) liquid flow in two parallel plates spinning in the same direction, with one of the plates permeable, in two parallel plates revolving in the same direction. They were able to develop a numerical solution and study the effects of numerous physical elements. They were successful. Recently, Reddy^37^ investigated MHD flow between binary rotating plates under the effect of heat radiation and H–H reactions in a binary rotating plate system. These references^38–41^ include more findings on rotating frame nanofluid flow with varied effects.

## Significance of this research

The principal objective of this work is to visualize and assess the fluid flow and the heat transfer analyses between the two parallel platters with a top plate rotating with convective boundary conditions for two distinct hybrid nanoliquids, which include cylindrical, platelet, and spherical particles. The entire system rotates uniformly in the specified direction. The variable uniform heat source-sink and linear thermal radiation effects are all incorporated in the thermal equation. Furthermore, multiple viscosity and thermal conductivity models are used based on shape properties. However, a review of the literature revealed that only a few experiments for the comparison of two hybrid nanofluids with distinct shape effects on channel flow had been undertaken. Furthermore, solar thermal absorption is important in storing energy in solar power plants, and solar systems commonly face the difficulty of storing and regulating energy at high temperatures. The goal is to discover how hybrid nanofluids may be used as solar energy absorbers while also having the capability of storing thermal energy as well as transferring it. The originality is that it presents the notion of a rotating top plate and compares the performance of two unique hybrid nanofluids, one with cylindrical platelet shape effects and another with cylindrical spherical shape effects, in solar thermal systems. While evaluating this investigation, the following questions will be addressed:

i. What effect would rotation have on the velocity and temperature profiles of two distinct hybrid nanofluids?
ii. Which type of Hybrid nanofluid is responsible for the increased heat in porous medium channels?
iii. How Slip parameter will affect the velocity profiles of hybrid nanofluids?
iv. What impact will the Biot number have on the temperature profile, and which hybrid nanofluid will generate the most heat?
v. How does radiative parameter affect temperature profile? Which hybrid nanofluid will have less absorption?
vi. What effect will the parameter for heat generation/absorption have on the temperature profile?
vii. Which Hybrid nanofluid with the highest performance in a solar thermal system has a combination of different shapes?

## Mathematical analysis

**Figure 1:**
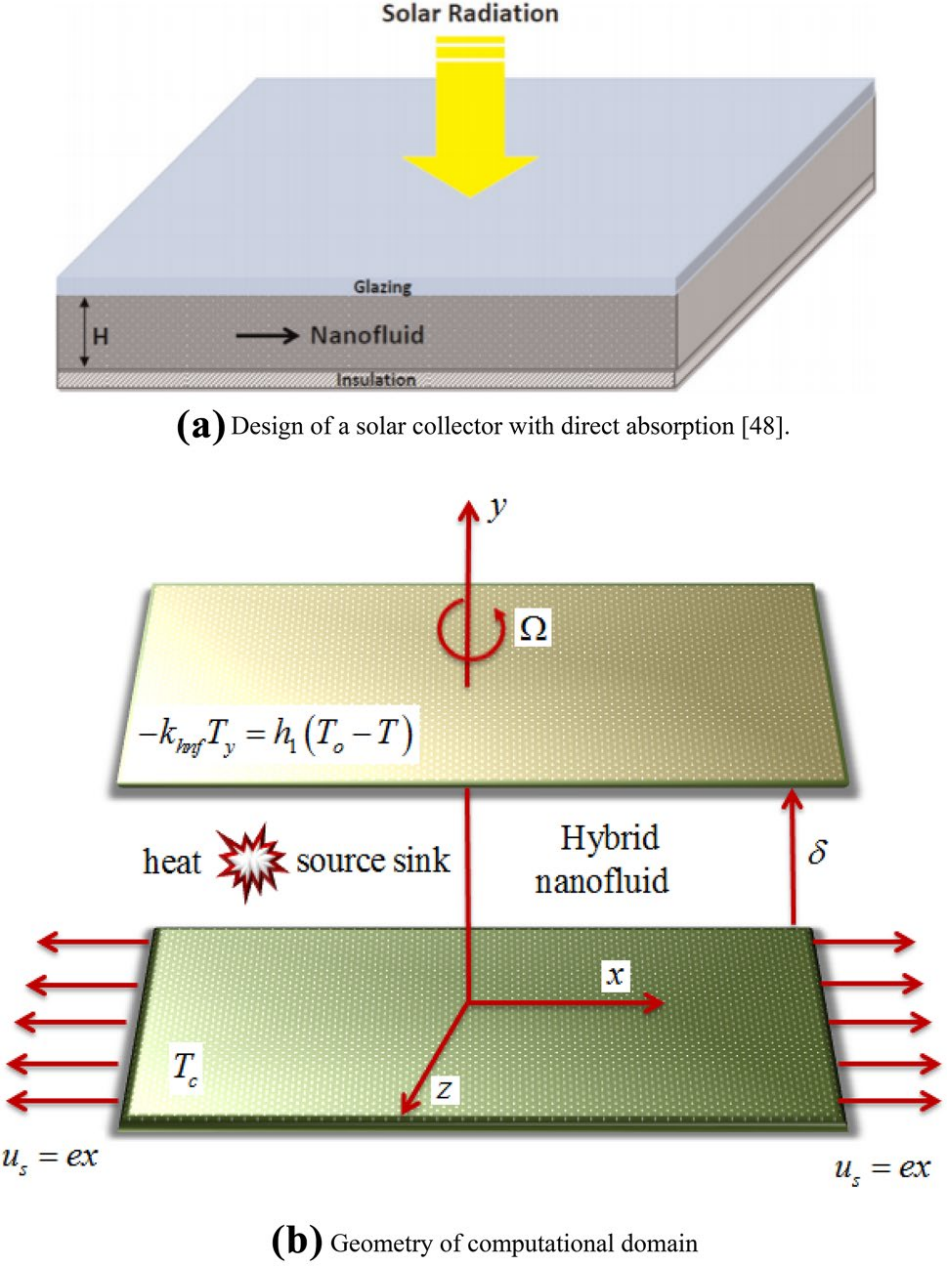
**(a)** Design of a solar collector with direct absorption^48^. **(b)** Geometry of computational domain.

The current study involves the three-dimensional flow of a steady, laminar and incompressible hybrid nanofluid confined by two parallel plates (horizontal) spaced $\delta$ apart in a rotating frame. The axis of the coordinate system is designed in a manner that both the plate and the liquid rotate about the $y$-axis with a constant angular velocity $\dot{\Omega }$. The bottom plate is extending linearly in the $x$-direction with a velocity $u_{s} = ex$ with $e > 0$. The top plate corresponds to both the slip and convective boundary conditions. Figure 1a,b are pictorial representation of schematic and computation diagrams respectively. Table 1 gives information about the thermo-physical traits of the customary fluid and the nanoparticles.

**Table 1:** Physical and thermal characteristics of water (working liquid), Graphene, Ag, and CuO (nanomaterials)^42–47^.

Working fluid/nanoparticles	$\left( \rho \right)$	$\left( {C_{p} } \right)$	$\left( k \right)$
Graphene (cylindrical)	2200	790	5000
Water	997.1	4179	0.6130
Ag (platelet)	10,500	235	429
CuO (spherical)	6500	535.6	20

The Tiwari das model with the following conservation equations is used to mathematically explain the flow of water-based various hybrid nanofluids with variable shape effects in a rotating frame using the following conservation equations:

1 $$ u_{x} + v_{y} = 0, $$

2 $$ uu_{x} + vu_{y} + 2\Omega w + \frac{{p_{x} }}{{\rho_{hnf} }} = \nu_{hnf} \left( {u_{xx} + u_{yy} } \right) - \frac{{\mu_{hnf} }}{{k^{ \bullet } \rho_{hnf} }}u, $$

3 $$ 0 = \frac{{p_{y} }}{{\rho_{hnf} }}, $$

4 $$ uw_{x} + vw_{y} - 2\Omega u = \nu_{hnf} \left( {w_{xx} + w_{yy} } \right) - \frac{{\mu_{hnf} }}{{k^{ \bullet } \rho_{hnf} }}w, $$

5 $$ \left( {\rho C_{p} } \right)_{hnf} \left( {uT_{x} + vT_{y} + wT_{z} } \right) = k_{hnf} \left( {T_{xx} + T_{yy} } \right) - \left( {q_{r} } \right)_{y} + Q^{ * } . $$

Net crossflow is experienced along $z$- axis, that’s why $p_{z}$ is absent from Eq. (4). Further, $qr$ and $Q^{ * }$ are radiative heat flux and heat source-sink respectively in Eq. (5), and are given as:

$$ qr = - \frac{{4\sigma^{*} }}{{3k^{ * } }}\frac{{\partial T^{4} }}{\partial y} = - \frac{{16\sigma^{*} T_{c}^{3} }}{{3k^{ * } }}\frac{\partial T}{{\partial y}}. $$

6 $$ Q^{ * } = \frac{{k_{hnf} u_{s} }}{{\nu_{hnf} x}}\left[ {Q_{o} \left( {T_{o} - T_{c} } \right)f^{\prime} + Q_{1} \left( {T - T_{c} } \right)} \right]. $$

The following are the boundary conditions for the scenario provided above:

$$ u = ex,\quad v = 0,\quad T = T_{c} \quad w = 0,\quad {\text{at}}\quad y = 0, $$

7 $$ u + \alpha_{1} \frac{\partial u}{{\partial y}} = 0,\quad v = 0,\quad - k_{hnf} \frac{\partial T}{{\partial y}} = h_{1} \left( {T_{o} - T} \right)\quad w = \alpha_{1} \frac{\partial w}{{\partial y}},\quad {\text{at}}\quad y = \delta . $$

## Simplification of mathematical analysis

Employing the following similarity transformations:

8 $$ u = exf^{\prime}\left( \eta \right),\quad v = - e\delta f\left( \eta \right),\quad w = exg\left( \eta \right),\quad \eta = \frac{y}{\delta },\quad \theta = \frac{{T - T_{c} }}{{T_{o} - T_{c} }}. $$

We obtain the following Ode’s by substituting Eq. (8) in Eqs. (1) to (7) after simplification and assuming pressure as $p_{xy} = p_{yx}$:

9 $$ f^{iv} + {\text{Re}}_{\delta } \frac{{\varepsilon_{b} }}{{\varepsilon_{a} }}\left( {ff^{\prime\prime\prime} - f^{\prime\prime}f^{\prime}} \right) - 2R_{o} \frac{{\varepsilon_{b} }}{{\varepsilon_{a} }}g^{\prime} - \lambda f^{\prime} = 0, $$

10 $$ g^{\prime\prime} + {\text{Re}}_{\delta } \frac{{\varepsilon_{b} }}{{\varepsilon_{a} }}\left( {fg^{\prime} - f^{\prime}g} \right) + 2R_{o} \frac{{\varepsilon_{b} }}{{\varepsilon_{a} }}f^{\prime} - \lambda g = 0, $$

11 $$ \theta^{\prime\prime}\left[ {\varepsilon_{c} + N_{r} } \right] + \varepsilon_{d} \Pr {\text{Re}}_{\delta } f\theta^{\prime} + \frac{{\varepsilon_{c} \varepsilon_{b} }}{{\varepsilon_{a} }}{\text{Re}}_{\delta } \left[ {Q_{o} f^{\prime} + Q_{1} \theta } \right] = 0, $$

With depicted conditions at boundaries:

12 $$ g\left( 0 \right) = 0,f\left( 1 \right) = 0,\theta \left( 0 \right) = 0,f\left( 0 \right) = 0,f^{\prime}\left( 0 \right) = 1,g\left( 1 \right) = - S_{1} g^{\prime}\left( 1 \right),f^{\prime}\left( 1 \right) = - S_{1} f^{\prime\prime}\left( 1 \right),\varepsilon_{c} \theta^{\prime}\left( 1 \right) + B_{i} \left( {1 + \theta \left( 1 \right)} \right) = 0. $$

Dimensionless parameters that result from the aforementioned equations are as follows:

13 $$ \Pr = \frac{{\mu_{f} \left( {C_{p} } \right)_{f} }}{{k_{f} }},{\text{Re}}_{\delta } = \frac{{e\delta^{2} \rho_{f} }}{{\mu_{f} }},N_{r} = \frac{{16\sigma^{*} T_{c}^{3} }}{{3k^{*} k_{f} }},{\text{R}}_{o} = \frac{{\Omega \delta^{2} \rho_{f} }}{{\mu_{f} }},\lambda = \frac{{\delta^{2} }}{{k^{ * } }},B_{i} = \frac{{h_{1} \delta }}{{k_{f} }},S_{1} = \frac{{\alpha_{1} }}{\delta },\varepsilon_{a} = \frac{{\mu_{hnf} }}{{\mu_{f} }},\varepsilon_{b} = \frac{{\rho_{hnf} }}{{\rho_{f} }},\varepsilon_{c} = \frac{{k_{hnf} }}{{k_{f} }},\varepsilon_{d} = \frac{{\left( {\rho C_{p} } \right)_{hnf} }}{{\left( {\rho C_{p} } \right)_{f} }}. $$

### Water-based hybrid nanofluids

Many researchers have created hybrid nanofluids with water as the foundation fluid and shown that they have superior thermal characteristics to water. Suresh et al.^49^ explored Al_2_O_3_-Cu/water hybrid nanofluids, whereas Nine et al.^50^ examined (Cu/CuO)/water hybrid nanofluids. Nanofluids comprised of silicon-MWCNTs and water were studied by Baghbanzadeh et al.^51^. CuTiO_2_/water hybrid nanoliquid was examined by Madhesh et al.^52^, whereas Ag-HEG-MWNT/water nanoliquid was researched by Baby and Ramaprabhu^53^.

### Thermal and physical models for hybrid nanofluid

The density $\left( \rho \right)_{hnf}$ and Heat capacity $\left( {\rho C_{p} } \right)_{hnf}$ of the hybrid nanofluid depending on particle shape are as follows^54–58^:

14 $$ \rho_{hnf} = \left( {1 - \phi_{a} - \phi_{b} } \right)\rho_{f} + \phi_{a} \rho_{a} + \phi_{b} \rho_{b} \to \left[ {\left( {\text{Spherical and Cylindrical}} \right){\text{ Hybrid}}} \right] $$

15 $$ \rho_{hnf} = \left( {1 - \phi_{c} - \phi_{b} } \right)\rho_{f} + \phi_{c} \rho_{c} + \phi_{b} \rho_{b} \to \left[ {\left( {\text{Platelet and Cylindrical}} \right){\text{ Hybrid}}} \right] $$

16 $$ \left( {\rho C_{p} } \right)_{hnf} = \left( {1 - \phi_{a} - \phi_{b} } \right)\left( {\rho C_{p} } \right)_{f} + \phi_{a} \left( {\rho C_{p} } \right)_{a} + \phi_{b} \left( {\rho C_{p} } \right)_{b} \to \left[ {\left( {\text{Spherical and Cylindrical}} \right){\text{ Hybrid}}} \right] $$

17 $$ \left( {\rho C_{p} } \right)_{hnf} = \left( {1 - \phi_{c} - \phi_{b} } \right)\left( {\rho C_{p} } \right)_{f} + \phi_{c} \left( {\rho C_{p} } \right)_{c} + \phi_{b} \left( {\rho C_{p} } \right)_{b} \to \left[ {\left( {\text{Platelet and Cylindrical}} \right){\text{ Hybrid}}} \right] $$

The following are the viscosity models of nanofluid with varied particle shapes^54,58^:

18 $$ \left( {\mu_{nf} } \right)_{a} = \mu_{f} \left( {1 + 2.5\phi + 6.2\phi^{2} } \right) \to \left( {\text{Spherical Particles}} \right) $$

19 $$ \left( {\mu_{nf} } \right)_{b} = \mu_{f} \left( {1 + 13.5\phi + 904.5\phi^{2} } \right) \to \left( {\text{Cylindrical Particles}} \right) $$

20 $$ \left( {\mu_{nf} } \right)_{c} = \mu_{f} \left( {1 + 37.1\phi + 612.6\phi^{2} } \right) \to \left( {\text{Platelet Particles}} \right) $$

The interpolation method may be used to estimate the effective dynamic viscosity of a hybrid nanofluid as:

22 $$ \mu_{hnf} = \frac{{\left( {\mu_{nf} } \right)_{a} \phi_{a} + \left( {\mu_{nf} } \right)_{b} \phi_{b} }}{\phi } \to \left[ {\left( {\text{Spherical and Cylindrical}} \right){\text{ Hybrid nanofluid}}} \right] $$

23 $$ \mu_{hnf} = \frac{{\left( {\mu_{nf} } \right)_{c} \phi_{c} + \left( {\mu_{nf} } \right)_{b} \phi_{b} }}{\phi } \to \left[ {\left( {\text{Platelet and Cylindrical}} \right){\text{ Hybrid nanofluid}}} \right] $$

Thermal conductivity of nanofluid obtained for varied shape particles^54,58^:

24 $$ \left( {k_{nf} } \right)_{a} = \frac{{k_{a} + 2k_{f} + 2\phi \left( {k_{a} - k_{f} } \right)}}{{k_{a} + 2k_{f} - \phi \left( {k_{a} - k_{f} } \right)}}k_{f} \to \left( {\text{Spherical Particles}} \right) $$

25 $$ \left( {k_{nf} } \right)_{b} = \frac{{k_{b} + 3.9k_{f} + 3.9\phi \left( {k_{b} - k_{f} } \right)}}{{k_{b} + 3.9k_{f} - \phi \left( {k_{b} - k_{f} } \right)}}k_{f} \to \left( {\text{Cylindrical Particles}} \right) $$

26 $$ \left( {k_{nf} } \right)_{c} = \frac{{k_{c} + 4.7k_{f} + 4.7\phi \left( {k_{c} - k_{f} } \right)}}{{k_{c} + 4.7k_{f} - \phi \left( {k_{c} - k_{f} } \right)}}k_{f} \to \left( {\text{Platelet Particles}} \right) $$

Similarly, the effective thermal conductivity of hybrid nanofluids including nanoparticles of multi-shapes may be calculated using the interpolation approach, which is described below

27 $$ k_{hnf} = \frac{{\left( {k_{nf} } \right)_{a} \phi_{a} + \left( {k_{nf} } \right)_{b} \phi_{b} }}{\phi } \to \left[ {\left( {\text{Spherical and Cylindrical}} \right){\text{ Hybrid nanofluid}}} \right] $$

28 $$ k_{hnf} = \frac{{\left( {k_{nf} } \right)_{c} \phi_{c} + \left( {k_{nf} } \right)_{b} \phi_{b} }}{\phi } \to \left[ {\left( {\text{Platelet and Cylindrical}} \right){\text{ Hybrid nanofluid}}} \right] $$

Above $\phi = \left( {\phi_{i} + \phi_{b} } \right)$, where (i = a, c) represent spherical and platelet particles respectively.

### Physical quantities

The dimensional expression of skin friction $C_{f}$ and $N_{u}$ Nusselt number are noted by:

29 $$ C_{f} = - \frac{{\tau_{w} }}{{\frac{1}{2}\rho_{hnf} \left( {ex} \right)^{2} }},{\text{where shear stress }}\tau_{w} = \mu_{hnf} \left. {u_{y} } \right|_{y = 0,\delta } , $$

30 $$ N_{u} = - \frac{{\delta q_{w} }}{{k_{f} \left( {T_{o} - T_{c} } \right)}},{\text{where heat flux}} q_{w} = - \left. {\left[ {k_{hnf} + \frac{{16\sigma^{ * } T_{c}^{3} }}{3k}} \right]T_{y} } \right|_{y = 0,\delta } , $$

Dimensionless expression of the above-mentioned quantities:

31 $$ \frac{\delta }{2}{\text{Re}}_{x} C_{f} = - \frac{{\varepsilon_{a} }}{{\varepsilon_{b} }}f^{\prime\prime}\left( 0 \right),Nu = - \theta^{\prime}\left( 0 \right)\left[ {\varepsilon_{c} + N_{r} } \right].\left( {\text{Lower plate}} \right) $$

32 $$ \frac{\delta }{2}{\text{Re}}_{x} C_{f} = - \frac{{\varepsilon_{a} }}{{\varepsilon_{b} }}f^{\prime\prime}\left( 1 \right),Nu = - \theta^{\prime}\left( 1 \right)\left[ {\varepsilon_{c} + N_{r} } \right]. \left( {\text{Upper plate}} \right) $$

Whence $Re_{x} = \frac{{ex^{2} }}{{\nu_{f} }},$ signifies the local Reynold number.

### Entropy generation analysis

The entropy generation for the hybrid nanofluid containing dissimilar shaped particles is formulated as:

33 $$ \dot{S}_{GEN} = \frac{{k_{hnf} }}{{T_{c}^{2} }}\left[ {1 + \frac{{16\sigma T_{c}^{3} }}{{3k_{f} k^{*} }}} \right]\left\{ {\left( {\frac{\partial T}{{\partial x}}} \right)^{2} + \left( {\frac{\partial T}{{\partial y}}} \right)^{2} } \right\} + \frac{{\mu_{hnf} }}{{k^{ * } T_{c} }}\left[ {u^{2} + w^{2} } \right], $$

After applying the transformation (Eq. 8) on Eq. (26), we obtain:

34 $$ Ns = \frac{{\dot{S}_{GEN} }}{{\dot{S}_{o} }} = \left[ {\left( {\varepsilon_{c} + N_{r} } \right)\theta^{{\prime}{2}} + \varepsilon_{a} Br\omega \lambda \left( {f^{{\prime}{2}} + g^{2} } \right)} \right], $$

where $\dot{S}_{{_{O} }} = \frac{{k_{f} \left( {T_{o} - T_{c} } \right)}}{{T_{c}^{2} \delta^{2} }},$ is the characteristics entropy generation.

## Numerical solution

**Figure 2:**
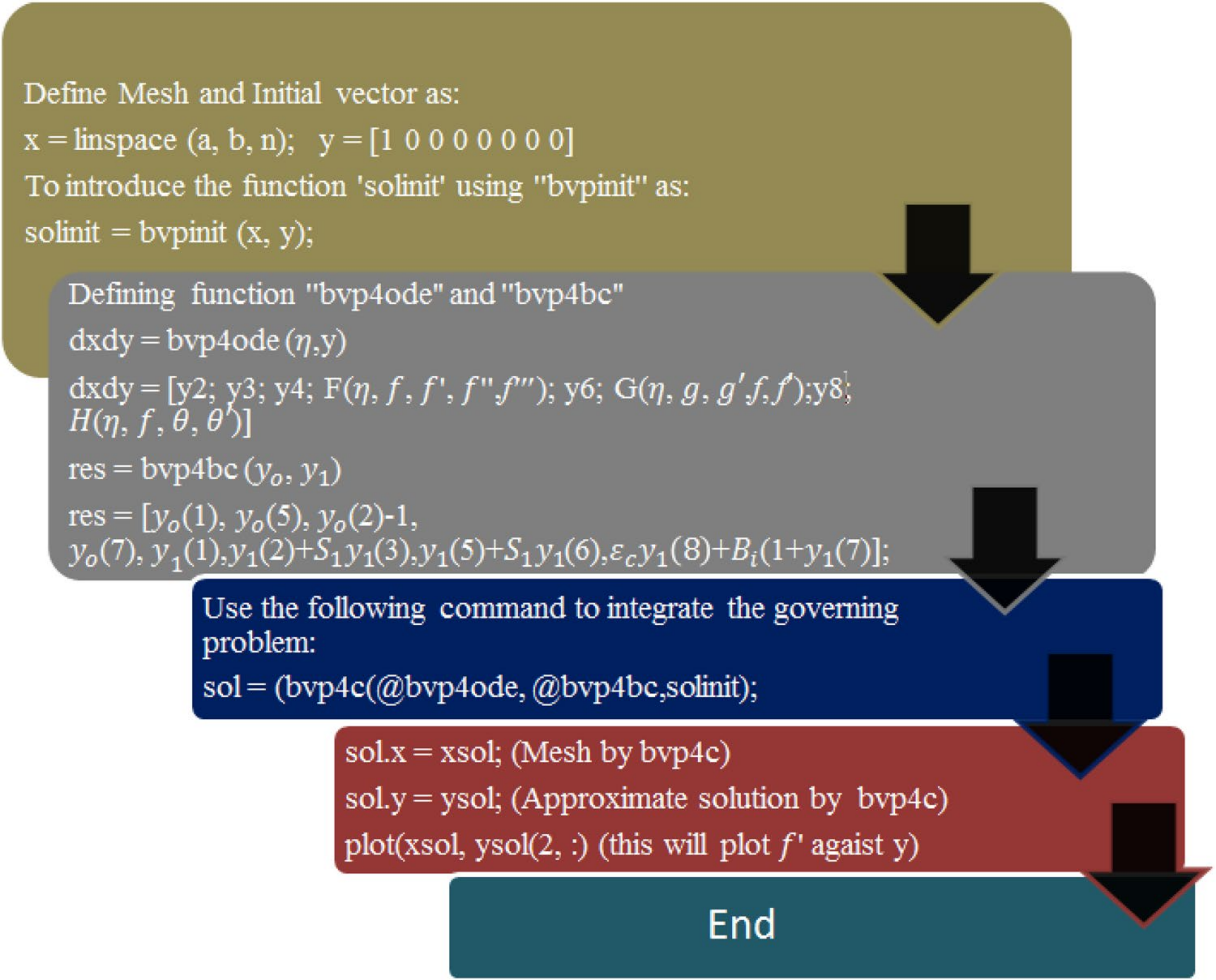
Flow chart of the algorithm.

The modified ordinary differential Eqs. (9)–(11), as well as the boundary conditions (12), are extremely nonlinear and analytically its solution is not possible; instead, the Bvp4c approach must be used to simplify them numerically. Bvp4c is a finite difference code that implements the three-stage Lobatto IIIa formula, which is a finite difference algorithm. This is a collocation formula, and the collocation polynomial yields a C1-continuous solution that is fourth-order accurate uniformly in [a, b] when applied to the collocation formula. The residual of the continuous solution is used to guide the selection of meshes and the management of errors. The flow chart of the algorithm is presented in Fig. 2. The model under examination has a tolerance of 10^–6^. Further, it is crucial that values that are finite of $\eta_{\infty }$ must be determined. For this computational objective, the asymptotic boundary conditions at $\eta_{\infty }$ for a given case are confined to $\eta = 1,$ which is required to illustrate the behavior of the required equations' asymptotic solution. To use the above computing approach, it is necessary to translate differential higher-order differential equations into differential equations of order one^59^. The numerical scheme's configuration is detailed below:

$$ \begin{gathered} y_{1} = f,y_{2} = f^{\prime},y_{3} = f^{\prime\prime},y_{4} = f^{\prime\prime\prime},yy_{1} = f^{iv} , \hfill \\ y_{5} = g,y_{6} = g^{\prime},yy_{2} = g^{\prime\prime}, \hfill \\ y_{7} = \theta ,y_{8} = \theta^{\prime},yy_{3} = \theta^{\prime\prime}, \hfill \\ yy_{1} = - {\text{Re}}_{\delta } \left( {\frac{{\varepsilon_{b} }}{{\varepsilon_{a} }}} \right)\left( {y_{1} y_{3} - y_{3} y_{2} } \right) + 2R_{o} \left( {\frac{{\varepsilon_{b} }}{{\varepsilon_{a} }}} \right)y_{6} + \lambda y_{2} , \hfill \\ yy_{2} = - {\text{Re}}_{\delta } \left( {\frac{{\varepsilon_{b} }}{{\varepsilon_{a} }}} \right)\left( {y_{1} y_{6} - y_{2} y_{5} } \right) - 2R_{o} \left( {\frac{{\varepsilon_{b} }}{{\varepsilon_{a} }}} \right)y_{2} - \lambda y_{5} , \hfill \\ yy_{3} = \left( {\frac{1}{{\varepsilon_{c} + N_{r} }}} \right)\left( { - \varepsilon_{d} \Pr {\text{Re}}_{\delta } y_{1} y_{8} - \left( {\frac{{\varepsilon_{c} \varepsilon_{b} }}{{\varepsilon_{a} }}} \right){\text{Re}}_{\delta } \left( {Q_{o} y_{2} + Q_{1} y_{5} } \right)} \right). \hfill \\ \end{gathered} $$

With associated boundary conditions:

$$y_{o} \left( 1 \right) = 0,$$ $$y_{1} \left( 1 \right) = 0,$$ $$y_{o} \left( 2 \right) - 1 = 0,$$ $$y_{1} \left( 2 \right) + S_{1} y_{1} \left( 3 \right) = 0,$$ $$y_{1} \left( 5 \right) + S_{1} y_{1} \left( 6 \right) = 0,$$ $$y_{o} \left( 7 \right) = 0,$$ $$y_{o} \left( 5 \right) = 0,$$ $$\varepsilon_{c} y_{1} \left( 8 \right) + B_{i} \left( {1 + y_{1} \left( 7 \right)} \right).$$

## Outcomes and discussion

**Figure 3:**
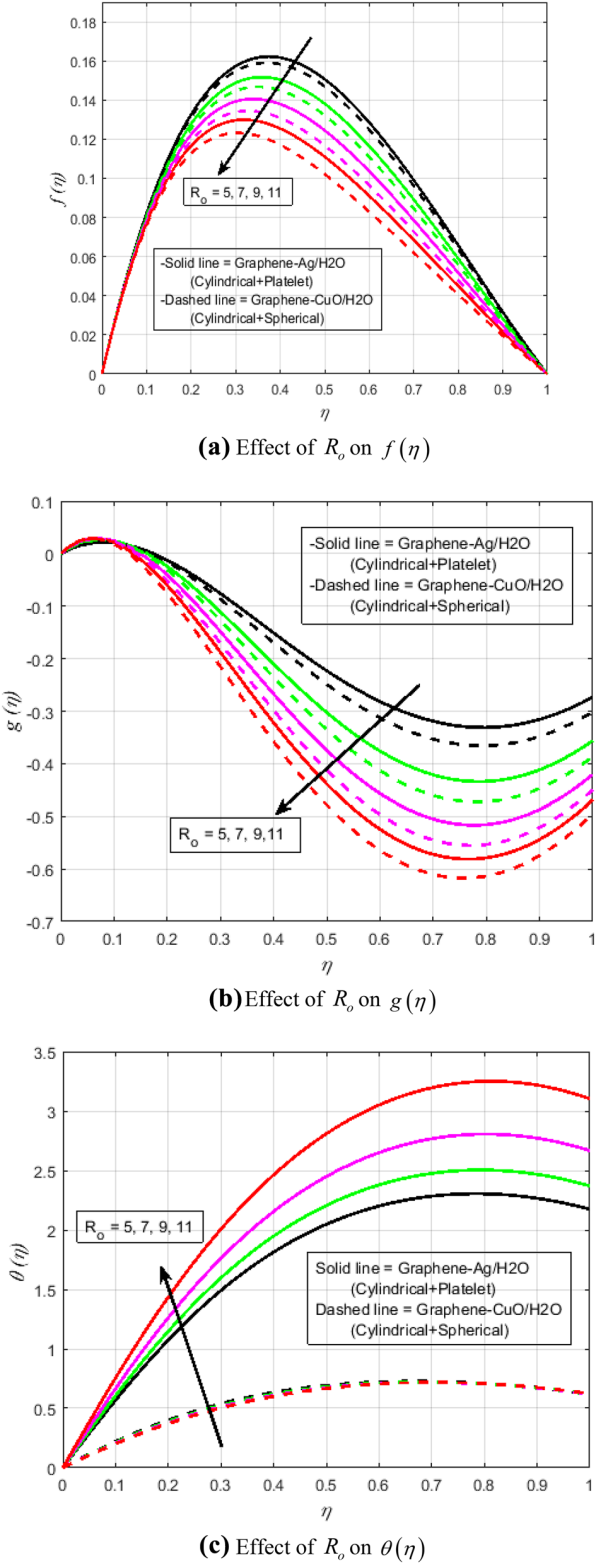
**(a)** Effect of $R_{o}$ on $f\left( \eta \right)$. **(b)** Effect of $R_{o}$ on $g\left( \eta \right)$. **(c)** Effect of $R_{o}$ on $\theta \left( \eta \right)$.

**Figure 4:**
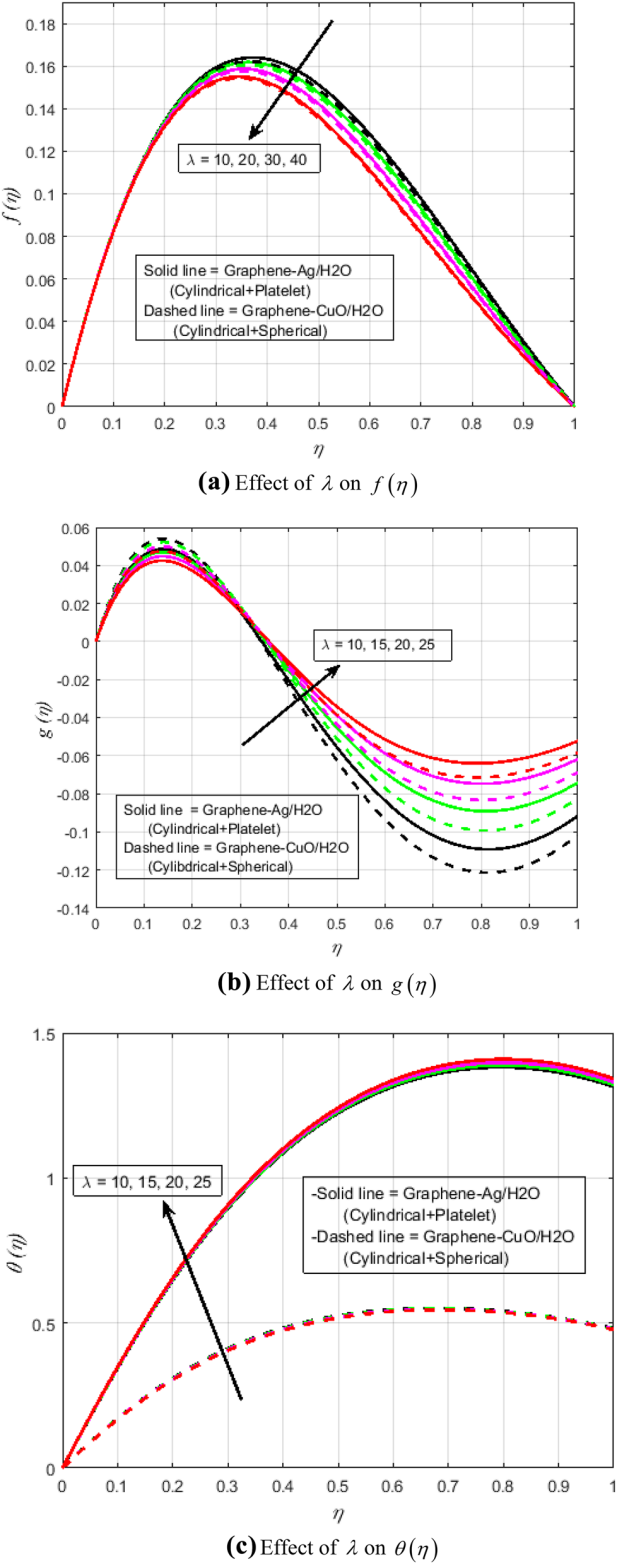
**(a)** Effect of $\lambda$ on $f\left( \eta \right)$. **(b)** Effect of $\lambda$ on $g\left( \eta \right)$. **(c)** Effect of $\lambda$ on $\theta \left( \eta \right)$.

**Figure 5:**
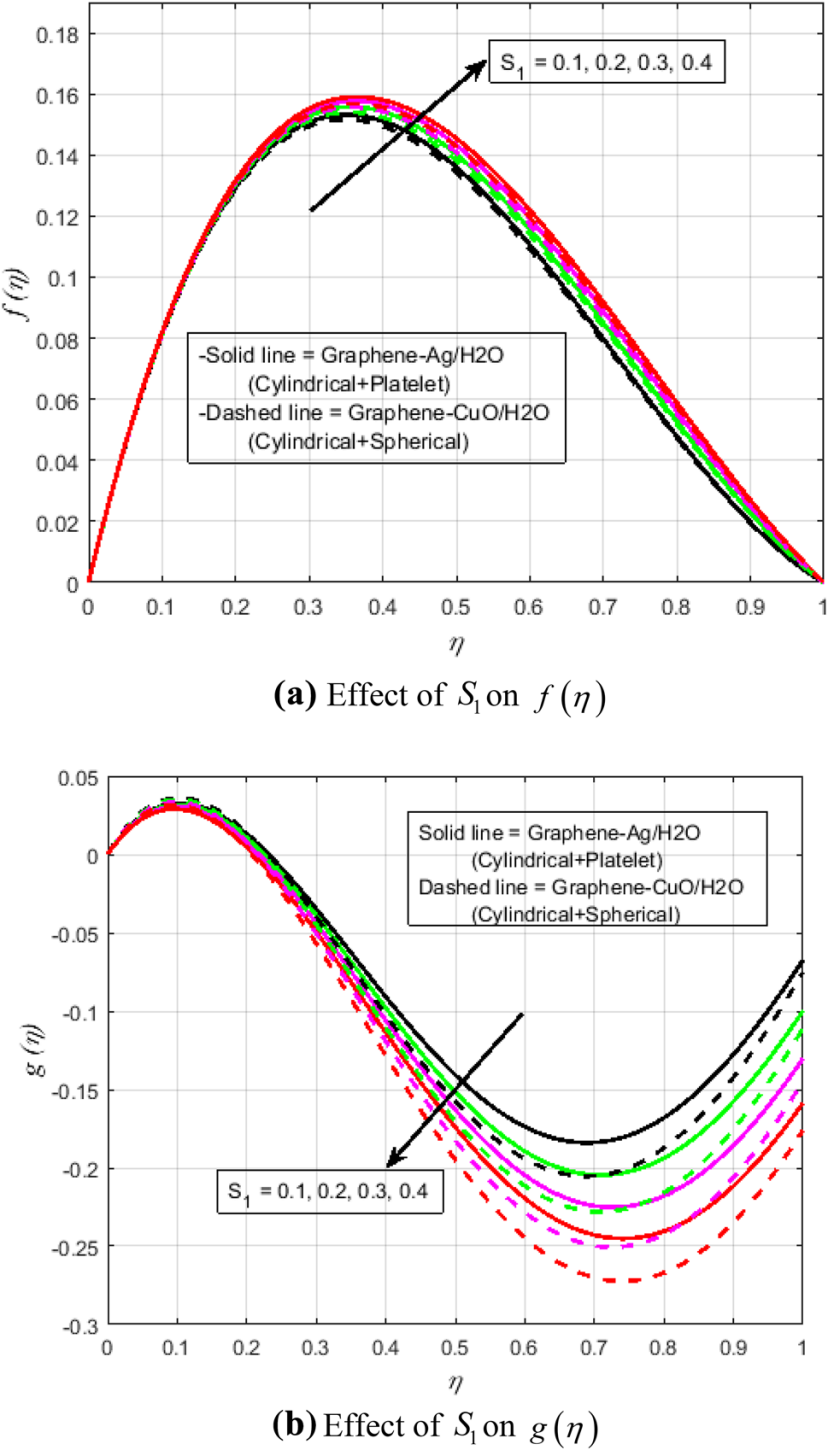
**(a)** Effect of $S_{1}$ on $f\left( \eta \right)$. **(b)** Effect of $S_{1}$ on $g\left( \eta \right)$.

**Figure 6:**
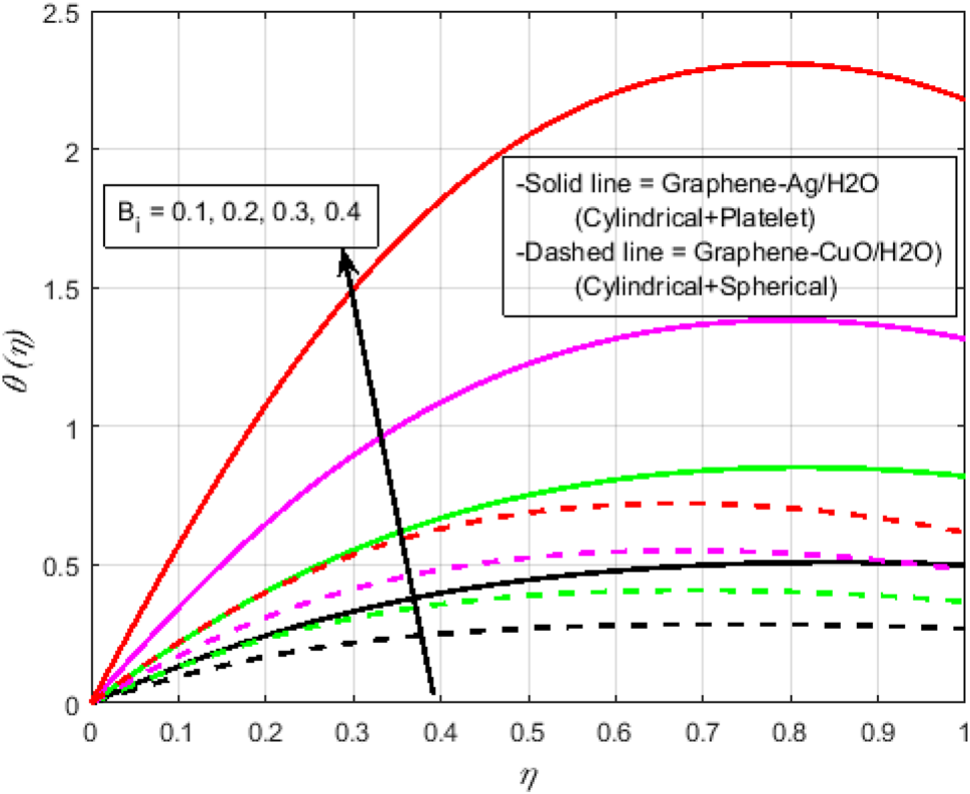
Effect of $B_{i}$ on $\theta \left( \eta \right)$.

**Figure 7:**
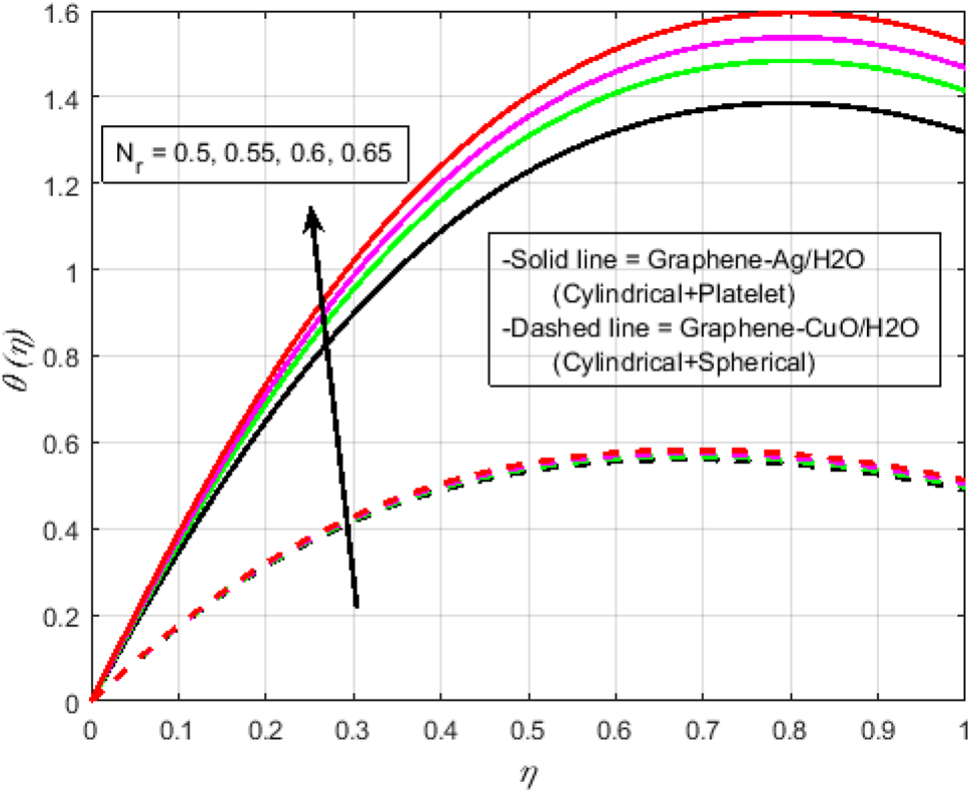
Effect of $N_{r}$ on $\theta \left( \eta \right)$.

**Figure 8:**
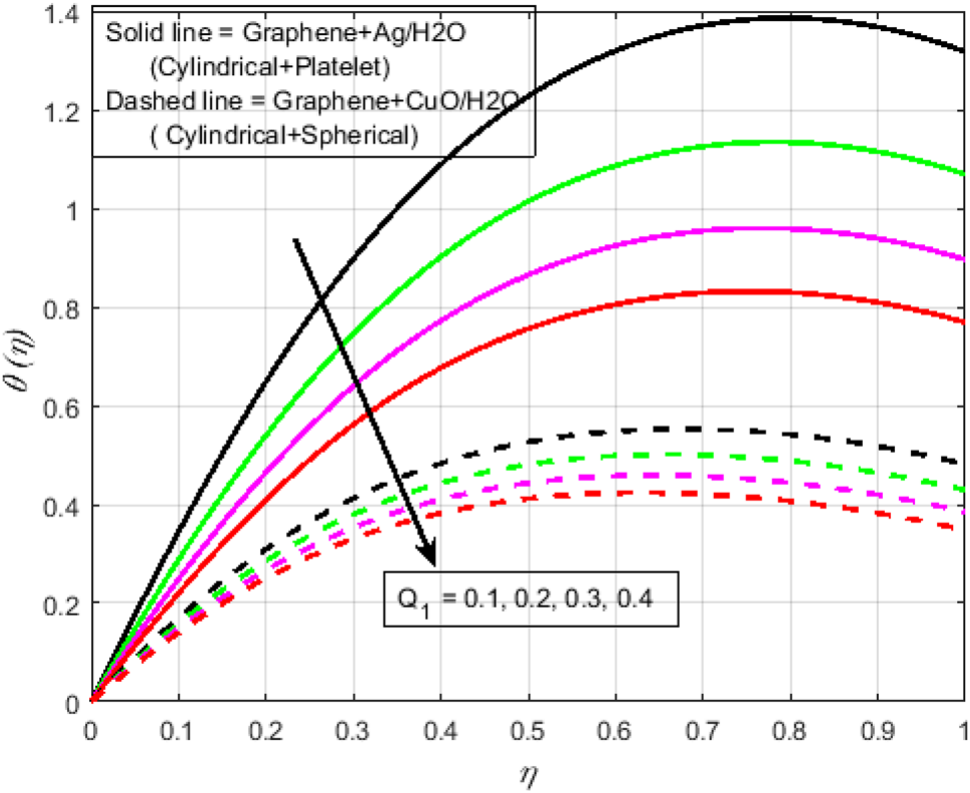
Effect of $Q_{1}$ on $\theta \left( \eta \right)$.

**Figure 9:**
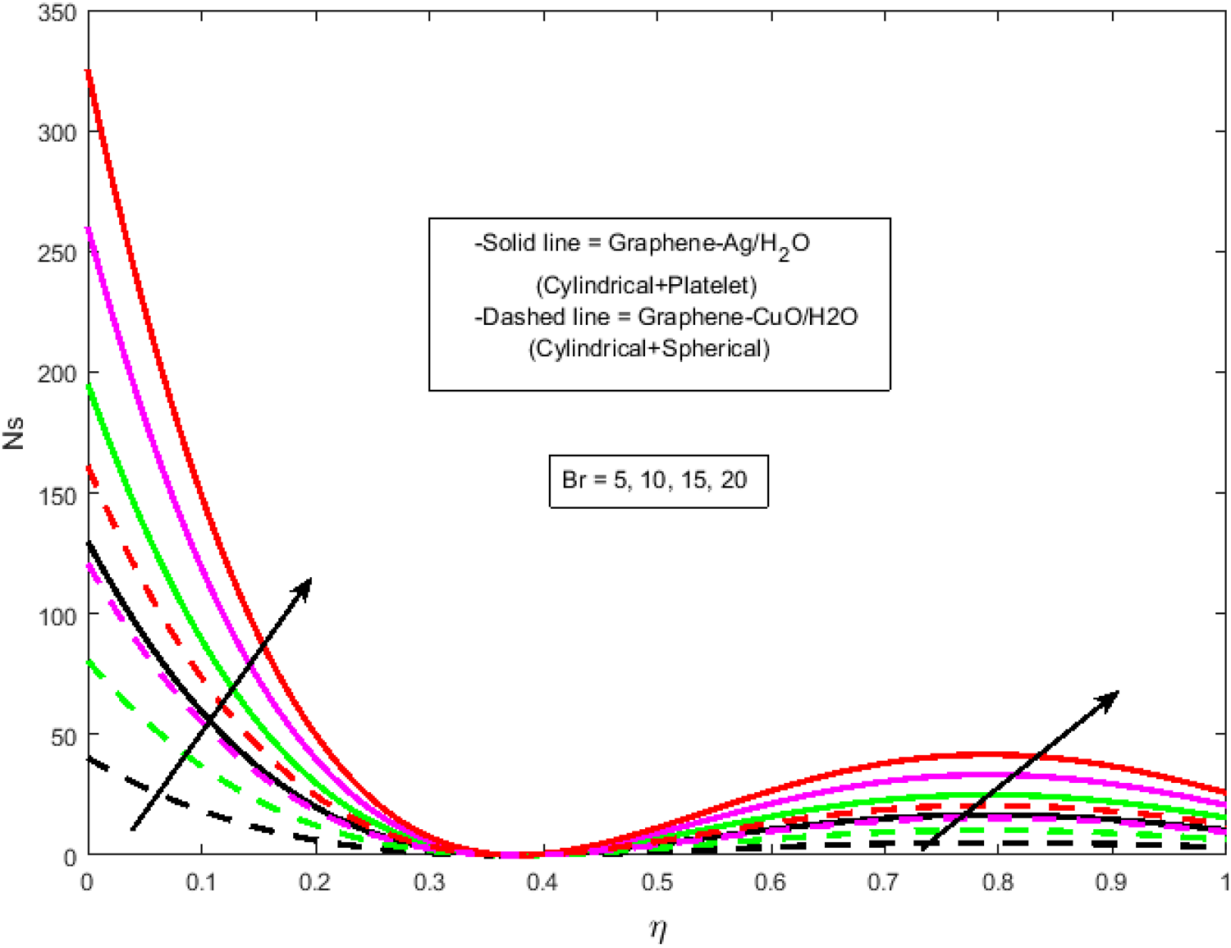
Effect of $Br$ on ${\text{Ns}}$.

**Figure 10:**
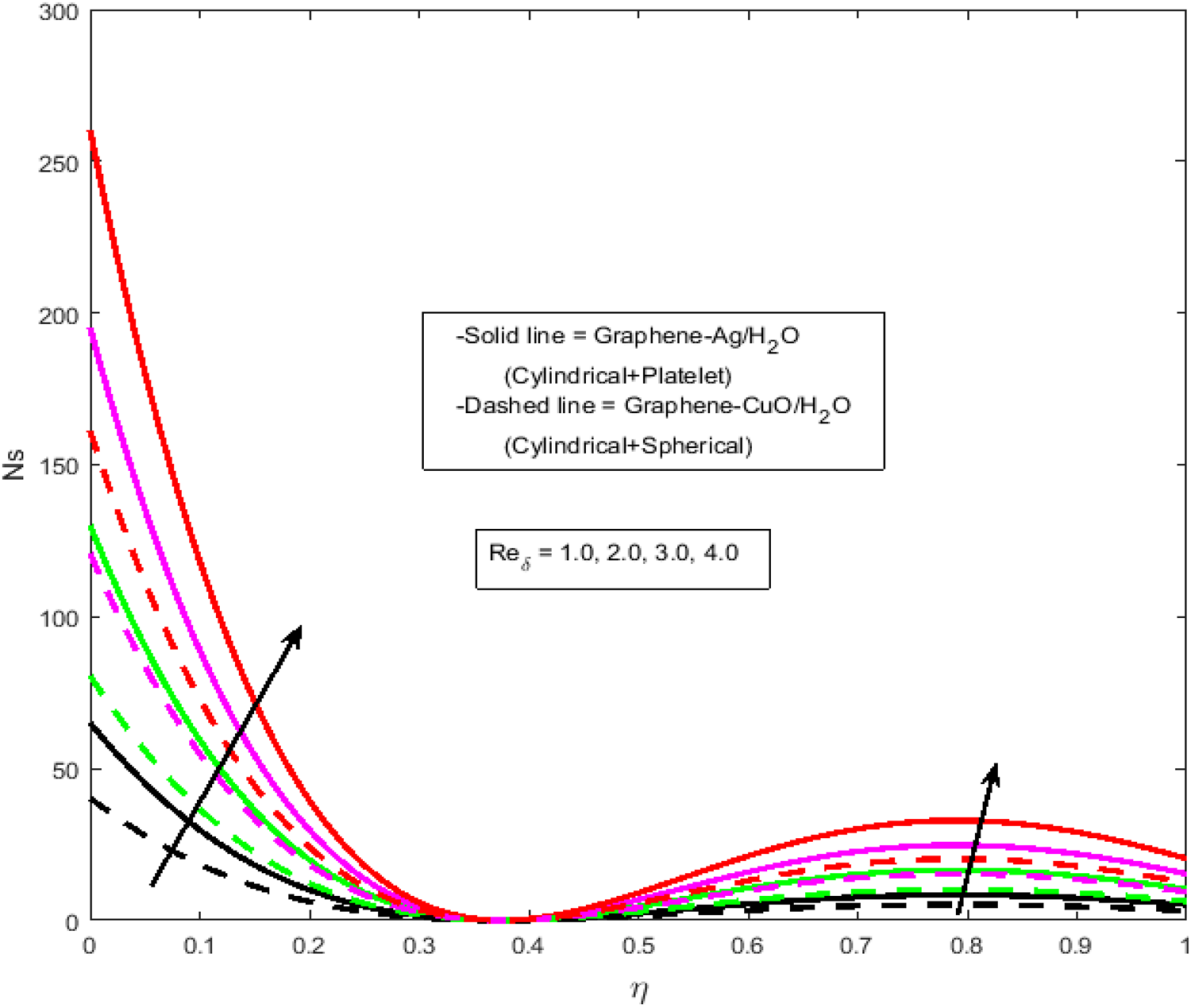
Effect of ${\text{Re}}_{\delta }$ on ${\text{Ns}}$.

Specific to this section's objectives is to assess the variations of various parameters as they are portrayed in graphical forms. The values employed to parameters used are $B_{i} = 0.3,Q_{o} = 0.01,Q_{1} = 0.02,\lambda = 10,\phi_{a} = 0.04,\phi_{b} = 0.05,\phi_{c} = 0.04,{\text{Re}}_{\delta } = 0.0003,R_{o} = 5,S_{1} = 0.4,$ $N_{r} = 0.5,\Pr = 6.2$. In all of the graphs, the comparison between two different hybrid nanofluids, Graphene-Ag/H_2_O and Graphene-CuO/H_2_O, which have different shape effects, is presented. Figure 3a–c are drawn for various values of rotation parameter $R_{o}$ on velocity and temperature profiles. The rotation parameter is a ratio of angular velocity to stretching rate. It is found that the primary velocity dwindled for mounting values of $R_{o}$, whereas secondary velocity first increases then decreases in the channel. This implies that rotation retards fluid flow in the primary flow direction and accelerates fluid flow in the secondary flow direction in the boundary layer region. This may be attributed to the fact that when the frictional layer at the moving plate is suddenly set into motion then the Coriolis force acts as a constraint in the main fluid flow *i.e.*, in the fluid flow in the primary flow direction to generate cross flow *i.e.*, secondary flow. The trend in the secondary profile is due to the rotation parameter for which the secondary profile oscillates in the middle of the channel that’s why both increasing and decreasing behaviors can be witnessed. Furthermore, the opposite trend can be seen for the temperature profile. This is due to increasing values of $R_{o}$ fluid velocity decrease which shows that there is more resistance for fluid flow as a result large amount of heat produces consequently temperature increases. It is also demonstrated in these three figures that the lowering velocity profile is more prominent for the Graphene-CuO/water hybrid nanofluid, whereas the increasing temperature profile is more prominent for the Graphene-Ag/water hybrid nanofluid. It's worth noting that without the rotation parameter $R_{o}$, the problem is reduced to a two-dimensional flow in a channel. Figure 4a–c are drawn for various values of $\lambda$ versus velocities and temperature profiles. As a result of raising the porosity parameter $\lambda ,$ primary velocity decreases while secondary velocity and temperature increase. This is because, when the porosity parameter of the fluid increases owing to an increase in its viscosity, a drop in its permeability at the edge, or a decrease in the stretching rate of the accelerating surface, the fluid's flow velocity gradually decreases which will further result in enhancement of temperature. The enhancement of temperature can be seen more for Graphene-Ag/water hybrid nanofluid. Figure 5a,b illustrate the effect of the slip parameter on velocity profiles. It is observed that as the slip parameter is increased, the primary velocity profile grows, while the secondary velocity profile decreases. This decrease is due to the fact that an increase in the slip factor generates the friction force which allows more fluid to slip past the sheet and the secondary flow decelerates. Figure 6 illustrates the influence of the Biot number on the temperature profile. Increased values of the $B_{i}$ number indicate that the plate's internal thermal resistance is greater than the plate's external thermal resistance. As a result, temperature increases with increasing $B_{i}$ levels. This increase in temperature is more noticeable in the case of Graphene-Ag/H_2_O. Figure 7 illustrates the effect of the radiation parameter $N_{r}$ on the temperature profile. For rising values of $N_{r}$, an increase is seen. As radiation parameter encounters the effects of transmission and absorption of the substance. An increase in the radiation parameter results in a drop in the mean absorption coefficient, which improves the temperature profile physically. Additionally, when the radiation parameter increases, the temperature gradient increases, resulting in an increase in fluid velocity. It is also noticed that the temperature increase is greater in the case of graphene-Ag/H_2_O hybrid nanofluid. This is crucial for solar thermal systems, which is necessary to warm the fluid that is operating in them. Figure 8 is drawn to visualize the impact of heat generation and absorption on the temperature profile. It can be seen that for the values of $Q_{1}$ less than zero, the temperature profile will reduce. Actually, for $Q_{1} < 0,$ the fluid absorbs heat from the thermal boundary layer resulting in a drop in the fluid's temperature. This pattern is more pronounced in the case of graphene-CuO/H_2_O hybrid nanofluid. Figure 9 is drawn to visualize the impact of the Brinkman number on entropy generation. The purpose of the inclusion of the Brinkman number is to analyze the rate of heat transfer from molecular conduction to viscous heating. It is also witnessed that entropy generation is more prominent for graphene-Ag/H_2_O hybrid nanofluid instead of graphene-CuO/H_2_O hybrid nanofluid. Further to visualize the impact of Reynold number against entropy profile Fig. 10 is sketched. Because of the enhancing impact of Reynolds number in all irreversibility processes, it is possible to see a rising trend with increasing values of Reynolds number over time. More heat is released from the nanofluid components to enhance entropy when the values of the Brinkman number escalate. Tables 2 and 3 illustrate the effect of various factors on skin friction and Nusselt number. As shown in Table 2, increasing the rotation $R_{o}$ and porosity parameters $\lambda$ results in an increase in the skin friction coefficient for both the upper and lower plate. This trend can be seen more for Graphene-Ag/H_2_O as compared to Graphene-CuO/H_2_O. From Table 4, it can be noticed that the Nusselt number enhances for higher values of radiation parameter $N_{r}$, for upper plate and reduces for the lower plate. Where for higher values of Reynolds number enhances at the lower wall but decreases at an upper wall for Graphene-Ag/H_2_O, while Nusselt’s number enhances for both upper and lower wall for Graphene-CuO/H_2_O. Table 4 is tabulated for grid analysis test against Nusselt number. It is noticed a grid size of 25 $\times$ 25 is enough for grid independence. Furthermore, for validation of the results of the presented study, a comparison with the published work is depicted in Table 5.

**Table 2:** Skin coefficient numerical values for rotational $R_{o}$ and porosity parameters $\lambda$.

$R_{o}$ (graphene-Ag/H_2_O)	Lower wall $f^{\prime\prime}\left( 0 \right)$	Upper wall $f^{\prime\prime}\left( 1 \right)$	$R_{o}$(graphene-CuO/H_2_O)	Lower wall $f^{\prime\prime}\left( 0 \right)$	Upper wall $f^{\prime\prime}\left( 1 \right)$
5	10.0510	−1.8350	5	8.8710	−1.5371
7	10.9110	−1.6690	7	9.7551	−1.3701
9	11.8900	−1.4920	9	10.7610	−1.2021
11	11.9912	−1.3241	11	11.8101	−1.0498

$\lambda$ (graphene-Ag/H_2_O)	Lower wall $f^{\prime\prime}\left( 0 \right)$	Upper wall $f^{\prime\prime}\left( 1 \right)$	$\lambda$(graphene-CuO/H_2_O)	Lower wall $f^{\prime\prime}\left( 0 \right)$	Upper wall $f^{\prime\prime}\left( 1 \right)$
10	9.6312	−1.7531	10	8.8710	−1.4820
15	9.5671	−1.6921	15	8.8610	−1.4340
20	9.5390	−1.6271	20	8.8490	−1.3820
25	9.5361	−1.5591	25	8.8390	−1.3260

**Table 3:** Nusselt number numerical values for radiation parameter $N_{r}$ and Reynolds number ${\text{Re}}_{\delta }$.

$N_{r}$ (graphene-Ag/H_2_O)	Lower wall $- \theta^{\prime}\left( 0 \right)$	Upper wall $\theta^{\prime}\left( 1 \right)$	$N_{r}$(graphene-CuO/H_2_O)	Lower wall $- \theta^{\prime}\left( 0 \right)$	Upper wall $\theta^{\prime}\left( 1 \right)$
0.5	1.9091	−1.8350	0.5	0.9564	0.2241
0.55	2.0031	−1.6690	0.55	0.9814	0.2295
0.6	2.1034	−1.4920	0.6	1.0071	0.2356
0.65	2.2192	−1.3241	0.65	1.0331	0.2406

${\text{Re}}_{\delta }$ (graphene-Ag/H_2_O)	Lower wall $- \theta^{\prime}\left( 0 \right)$	Upper wall $\theta^{\prime}\left( 1 \right)$	${\text{Re}}_{\delta }$(graphene-CuO/H_2_O)	Lower wall $- \theta^{\prime}\left( 0 \right)$	Upper wall $\theta^{\prime}\left( 1 \right)$
0.0003	2.2156	0.3972	0.0003	1.0331	0.2406
0.0004	1.0741	0.2404	0.0004	0.8836	0.2057
0.0005	0.9274	0.2105	0.0005	0.9161	0.1933
0.0006	0.9235	0.1982	0.0006	1.0081	0.1871

**Table 4:** Analysis of grid points for the Nusselt number as follows.

S. #	Grid size	$Nu$
1	5 $\times$ 5	−0.3393
2	10 $\times$ 10	−0.3359
3	15 $\times$ 15	−0.3360
4	20 $\times$ 20	−0.3360
5	25 $\times$ 25	−0.3360
6	30 $\times$ 30	−0.3360

**Table 5:** Comparative analysis of Nusselt number for different values of Prandtl number when remaining parameters of temperature equation are zero.

$\Pr$	Xia et al.^60^	Ishak et al.^61^	Present
1	0.809	0.806	0.809
2	1.000	1.000	1.001
3	1.924	1.923	1.925
4	3.721	3.720	3.723

## Concluding remarks

The current study involves the three-dimensional flow of a steady, laminar and incompressible convective hybrid nanofluid confined by two parallel plates (horizontal) spaced $\delta$ apart in a rotating frame. In this work, two different hybrid nanofluids are examined with dissimilar shapes. Additionally, the effects of various factors on various profiles are represented and shown. The situation's significant repercussions are outlined below:

- A rotational parameter $R_{o}$ has declined the velocity profiles but enhanced the temperature profile. And the decline effect is significant in the case of Graphene-CuO/H_2_O whereas the enhancement effect of temperature is significant for Graphene-Ag/H_2_O.
- In porous media, Graphene-Ag/H_2_O is significant for the enhancement of temperature.
- The slip parameter enhances the primary velocity and reduces the secondary velocity.
- By increasing the biot number temperature profiles enhances. And this effect is significant in Graphene-Ag/water.
- By increasing the radiation parameter temperature profile enhances and this effect is significant in the case of Graphene-Ag/Hybrid nanofluid.
- The entropy profile enhances when the Brinkman number escalates to higher and higher levels. The quantity of heat discharge in Graphene-Ag/H_2_O is larger than in Graphene-CuO/H_2_O. As a result of the increased irreversibility of Graphene-Ag/H_2_O, it will be ineffective in solar thermal systems.
- Temperature profile decline for values of heat generation and absorption less than zero. And this effect is more pronounced in the case of Graphene-CuO hybrid nanofluid.
- More heat loss is witnessed for graphene-CuO/H_2_O than graphene-Ag/H_2_O. As a result, the solar system must be able to collect more heat than it emits. Graphene-Ag/H_2_O hybrid nanofluid with cylindrical and platelet particles works better in solar thermal energy systems than a mixture of cylindrical and spherical shape particles.

## List of Symbols

$k_{hnf}$: Thermal conductivity of hybrid nanofluid $\left( {{\text{W\,K}}^{ - 1} {\text{m}}^{ - 1} } \right)$
$$u,v,w$$: Velocity components $$\left( {{\text{m\,s}}^{ - 1} } \right)$$
$N_{r}$: Thermal radiation parameter
$\Omega$: Constant angular velocity $\left( {{\text{m\,s}}^{ - 1} } \right)$
$$T$$: Temperature $$\left( {\text{K}} \right)$$
$$\Pr$$: Prandtl number
$\mu_{hnf}$: Dynamic viscosity of hybrid nanofluid
$\left( {C_{p} } \right)_{f}$: Effective heat capacity of hybrid nanofluid $\left( {{\text{J}}{\kern 1pt} {\text{kg}}^{ - 1}\, {\text{K}}^{ - 1} } \right)$
$T_{c}$: Lower plate temperature
$g\left( \eta \right)$: Dimensionless secondary velocity
$q_{r}$: Radiative heat flux $\left( {{\text{W\,m}}^{ - 2} } \right)$
$$\mu$$: Dynamic viscosity $$\left( {{\text{kg}}{\kern 1pt} {\text{m}}^{ - 1} {\text{s}}^{ - 1} } \right)$$
$$x,y,z$$: Coordinates axis $$\left( {\text{m}} \right)$$
$$\nu$$: Kinematic viscosity $$\left( {{\text{m}}^{2}\, {\text{s}}^{ - 1} } \right)$$
$\left( {C_{p} } \right)_{nf}$: Effective heat capacity of nanofluid
$h_{1}$: Heat transfer coefficient
$\rho_{hnf}$: Density of hybrid nanofluid $\left( {{\text{kg}}\;{\text{m}}^{ - 3} } \right)$
$$R_{o}$$: Rotation parameter
$a,b,c$: Subscripts for spherical, cylindrical, and platelet
$\rho_{s}$: Density of particle
${\text{Re}}_{x}$: Local Reynold number
$\delta$: Distance between the plates
$\rho_{nf}$: Density of nanofluid $\left( {{\text{kg}}{\kern 1pt} {\text{m}}^{ - 3} } \right)$
$f\left( \eta \right)$: Dimensionless primary velocity
$\phi$: Particle volume fraction
$$Br$$: Brinkman number
$k^{ \bullet }$: Permeability of porous medium
$\mu_{nf}$: Dynamic viscosity of nanofluid
$k_{f}$: Thermal conductivity of the fluid $\left( {{\text{W\,K}}^{ - 1} {\text{m}}^{ - 1} } \right)$
$Q_{0} ,Q_{1}$: Nonuniform heat source and sink parameters
$$C_{f}$$: Skin friction
$$N_{u}$$: Nusselt number
$\left( {C_{p} } \right)_{f}$: Effective heat capacity of fluid $\left( {{\text{J\,kg}}^{ - 1} {\text{K}}^{ - 1} } \right)$
$e$: Stretching rate of the lower plate
$T_{o}$: Upper wall temperature
$$p$$: Pressure $$\left( {{\text{Pa}}} \right)$$
$\alpha_{nf}$: Thermal diffusivity of nanofluid
$\nu_{hnf}$: Kinematic viscosity of hybrid nanofluid
$$\eta$$: Similarity variable
$\nu_{nf}$: Kinematic viscosity of nanofluid
$k^{*}$: Mean absorption coefficient
$\rho_{f}$: Density of working fluid
$$B_{i}$$: Biot number
$\sigma^{ * }$: Stefan–Boltzmann constant $\left( {{\text{kg/s}}^{{3}} {\text{K}}^{{4}} } \right)$
$\alpha_{f}$: Thermal diffusivity fluid
$$\theta \left( \eta \right)$$: Dimensionless temperature
$$q_{w}$$: Heat flux
$$\lambda$$: Porosity parameter
$k_{nf}$: Thermal conductivity of nanofluid $\left( {{\text{W\,K}}^{ - 1} {\text{m}}^{ - 1} } \right)$
$\tau_{w}$: Wall shear stress
$${\text{Re}}_{\delta }$$: Reynold number
$\omega$: Non-dimensional temperature difference
